# The voltage gated Ca^2+^-channel Ca_v_3.2 and therapeutic responses in breast cancer

**DOI:** 10.1186/s12935-016-0299-0

**Published:** 2016-03-31

**Authors:** Elena Pera, Elke Kaemmerer, Michael J. G. Milevskiy, Kunsala T. D. S. Yapa, Jake S. O’Donnell, Melissa A. Brown, Fiona Simpson, Amelia A. Peters, Sarah J. Roberts-Thomson, Gregory R. Monteith

**Affiliations:** The School of Pharmacy, Pharmacy Australia Centre of Excellence, The University of Queensland, 20 Cornwall St, Woolloongabba, Brisbane, QLD Australia; Mater Research Institute, The University of Queensland, Brisbane, QLD Australia; Translational Research Institute, Brisbane, QLD Australia; The School of Chemistry and Molecular Biosciences, The University of Queensland, Brisbane, QLD Australia; Diamantina Institute, The University of Queensland, Brisbane, QLD Australia

**Keywords:** Breast cancer, Trastuzumab-resistance, Calcium-signalling, Ca_v_3.2 (*CACNA1H*), Therapeutic response, Biomarker

## Abstract

**Background:**

Understanding the cause of therapeutic resistance and identifying new biomarkers in breast cancer to predict therapeutic responses will help optimise patient care. Calcium (Ca^2+^)-signalling is important in a variety of processes associated with tumour progression, including breast cancer cell migration and proliferation. Ca^2+^-signalling is also linked to the acquisition of multidrug resistance. This study aimed to assess the expression level of proteins involved in Ca^2+^-signalling in an in vitro model of trastuzumab-resistance and to assess the ability of identified targets to reverse resistance and/or act as potential biomarkers for prognosis or therapy outcome.

**Methods:**

Expression levels of a panel of Ca^2+^-pumps, channels and channel regulators were assessed using RT-qPCR in resistant and sensitive age-matched SKBR3 breast cancer cells, established through continuous culture in the absence or presence of trastuzumab. The role of Ca_v_3.2 in the acquisition of trastuzumab-resistance was assessed through pharmacological inhibition and induced overexpression. Levels of Ca_v_3.2 were assessed in a panel of non-malignant and malignant breast cell lines using RT-qPCR and in patient samples representing different molecular subtypes (PAM50 cohort). Patient survival was also assessed in samples stratified by Ca_v_3.2 expression (METABRIC and KM-Plotter cohort).

**Results:**

Increased mRNA of Ca_v_3.2 was a feature of both acquired and intrinsic trastuzumab-resistant SKBR3 cells. However, pharmacological inhibition of Ca_v_3.2 did not restore trastuzumab-sensitivity nor did Ca_v_3.2 overexpression induce the expression of markers associated with resistance, suggesting that Ca_v_3.2 is not a driver of trastuzumab-resistance. Ca_v_3.2 levels were significantly higher in luminal A, luminal B and HER2-enriched subtypes compared to the basal subtype. High levels of Ca_v_3.2 were associated with poor outcome in patients with oestrogen receptor positive (ER+) breast cancers, whereas Ca_v_3.2 levels were correlated positively with patient survival after chemotherapy in patients with HER2-positive breast cancers.

**Conclusion:**

Our study identified elevated levels of Ca_v_3.2 in trastuzumab-resistant SKBR3 cell lines. Although not a regulator of trastuzumab-resistance in HER2-positive breast cancer cells, Ca_v_3.2 may be a potential differential biomarker for survival and treatment response in specific breast cancer subtypes. These studies add to the complex and diverse role of Ca^2+^-signalling in breast cancer progression and treatment.

**Electronic supplementary material:**

The online version of this article (doi:10.1186/s12935-016-0299-0) contains supplementary material, which is available to authorized users.

## Background

Approximately 20–25 % of breast tumours display overexpression of human epidermal growth factor receptor 2 (HER2, *ERBB2*) [1]. The monoclonal antibody trastuzumab (Herceptin^®^) represents a major advancement in the treatment of HER2-positive breast cancers [2, 3]. However, some patients can develop resistance to trastuzumab therapy [4, 5] while others exhibit initial therapeutic insensitivity, despite their tumours being identified as HER2-positive and the patients being naïve to trastuzumab therapy (intrinsic resistance) [6, 7].

Overcoming therapeutic resistance is a major challenge and an understanding of the underlying mechanism will inform the development of second generation treatments [8]. For example, resistance to tyrosine kinase inhibitors in lung cancer is attributed to the acquisition of an EGFR T790M mutation, and evidence suggests that a combination of irreversible tyrosine kinase inhibitors and an anti-EGFR antibody is effective in overcoming this particular resistance mechanism [9].

Different mechanisms may be responsible for acquired and intrinsic trastuzumab-resistance. These mechanisms include, but are not limited to, mutations in *ERBB2* resulting in the expression of a modified HER2 receptor altering trastuzumab binding [10, 11] and upregulation of proteins that sterically hinder trastuzumab binding [12, 13]. Increased signalling through HER1, HER2, HER3 (receptors of the EGFR-family) and IGF-1R [14–17] as well as downstream signalling such as activation of the PTEN/PI3K/Akt pathway also represent potential pathways for trastuzumab-resistance [7, 18–20]. Many of the aforementioned studies have been evaluated in breast cancer cell lines established from HER2-positive breast cancer cells cultured in the presence of trastuzumab, including the HER2-positive SKBR3 cell line [15, 16, 21].

A remodelling of Ca^2+^-signalling occurs in some breast cancers and is thought to be an important contributor or biomarker of breast tumourigenesis [22]. For example, enhanced expression of the Ca^2+^-channel TRPV6 is a feature of oestrogen receptor negative breast cancers [23] and alteration in the relative levels of the store operated Ca^2+^-influx pathway regulators STIM1 and STIM2 are a feature of the basal molecular breast cancer subtype and is associated with poor survival [24]. Ca^2+^ is a critical regulator of many processes important in cancer [25], including proliferation and migration [26, 27]. Indeed, inhibition of the Orai1 Ca^2+^-channel reduces the metastatic potential of breast cancer cells [28].

Ca^2+^-signalling is also implicated in some therapeutic resistance pathways in breast cancer. For example, the Ca^2+^-permeable ion channel TRPC5 plays a role in p-glycoprotein-mediated resistance to adriamycin in MCF-7 breast cancer cells [29]. However, the potential contribution of remodelling of Ca^2+^-signalling in trastuzumab-resistance has not yet been explored. Herein we sought to determine alterations of Ca^2+^-signalling proteins in the context of trastuzumab-resistance using HER2-positive SKBR3 breast cancer cell lines as models of intrinsic (no previous trastuzumab exposure) and acquired resistance. This work had the goal of identifying calcium channels and pumps that when inhibited could restore sensitivity to therapy and/or serve as biomarkers for prognosis or response to therapy.

## Methods

### Cell culture and development of resistant cell lines

Human breast cell lines were purchased from ATCC, provided by UQCCR or were a gift from the late Professor Rob Sutherland (Garvan Institute, Sydney, Australia). SKBR3 cells were subcultured in McCoy’s 5A media (Invitrogen) supplemented with 10 % foetal bovine serum and 1 % penicillin/streptomycin mixture (100 U/mL/100 µg/mL, Invitrogen) at 37 °C and 5 % CO_2_. Cells were routinely tested for mycoplasma infection and the SKBR3 parental cell line was STR profiled as previously described [30]. Trastuzumab-resistant cell lines were developed as follows, adapted from [31]. Briefly, cells were cultured in the presence of trastuzumab (10 µg/mL, Herceptin^®^, Roche Products, Dee Why, Australia) over a 7 month period. Trastuzumab treatment was initiated 24 h after seeding. Age-matched controls (no trastuzumab) were produced over a similar time period. Media (±trastuzumab) was replaced every 3 days.

### Cell viability, MTS assay

Cell viability was assessed using the CellTiter 96^®^ aqueous non-radioactive cell proliferation assay (Promega) using the manufacturer’s instructions. Cell lines were treated with trastuzumab or control media without antibiotics.

### Real time RT-qPCR (RT-qPCR)

Real time RT-qPCR (RT-qPCR) was used to assess mRNA levels of target genes as previously described [32]. Briefly, total RNA (Qiagen RNeasy™ Plus Mini Kit (Qiagen, Hilden, Germany) was reverse transcribed (Ominiscript RT Kit Qiagen) and amplified using TaqMan Universal or TaqMan Fast Universal PCR master mix (Life technologies, Australia), with TaqMan Gene expression Assays (Additional file 1: Data S1). Experiments were performed using a StepOnePlus Real Time RT-qPCR instrument (Applied Biosystems, Carlsbad, USA) with universal cycling conditions. Results are expressed normalised to 18S rRNA and analysed using the comparative C_T_ method. A C_T_ value of 35 was assigned to samples where amplification did not occur within 40 cycles for characterisation of trastuzumab-resistant and aged-matched control SKBR3 cell lines and assessment of Ca_v_3.2 levels in breast cancer cell lines (Taqman Universal PCR master mix). A C_T_ of 40 was assigned to samples where amplification did not occur within 40 cycles for studies assessing potential mRNA changes induced by Ca_v_3.2 overexpression (TaqMan Fast PCR master mix).

### Immunoblotting

Whole cell lysates were prepared using lysis buffer containing protease and phosphatase inhibitors (Roche, Applied Science, Penzberg, Germany). Proteins were separated using 4–12 % NuPAGE Bis–Tris gels with MOPS SDS running buffer (Invitrogen) and transferred to a PVDF membrane. Membranes were blocked in phosphate buffered saline (PBS)/1 %Tween20 or in PBS/5 % skim milk powder for 1 h. Antibodies included: HER2 polyclonal rabbit (Tyr 1222, Cell Signaling), EGFR polyclonal rabbit (Tyr 992, Cell Signaling), β-actin loading control (AC-15, Sigma Aldrich) and horseradish peroxidase conjugated goat anti-rabbit IgG (170-6516, Biorad). Membranes were incubated with SuperSignal West Dura Extended Duration Substrate (Thermo Scientific) and images analysed using a Versadoc MP400 Imaging system (BioRad). Protein density was normalised to β-actin.

### Pharmacological inhibition of Ca_v_3.2

Cells were treated with mibefradil (0.01–1 µM; Sigma Aldrich) [33] or ML218 (0.1–10 µM; Sigma Aldrich) [34] either alone or in combination with trastuzumab (10 µg/mL), 24 h after seeding (2000 cells/well in a 96 well plate). All treatments and media changes were repeated every 2 days in antibiotic-free media. Sensitivity to trastuzumab was assessed using a MTS assay 192 h after treatment start.

### Measurement of cytosolic free calcium levels

Cytosolic free Ca^2+^ ([Ca^2+^]_CYT_) levels were measured using a fluorometric imaging plate reader (FLIPR^TETRA^, Molecular Devices, Sunnyvale, USA). Cells (2000 cells/well in a 96-well black-walled imaging plate; Corning) were assessed 216 h after seeding in antibiotic-free media. Cells were treated with Fluo-4 AM (4 µM) for 30 min at 37 °C, washed three times in physiological salt solution (PSS) buffer [NaCl (140 mM)], glucose (11.5 mM), CaCl_2_ (1.8 mM), HEPES (10 mM), KCl (5.9 mM), MgCl_2_ (1.4 mM), NaH_2_PO_4_ (1.2 mM), NaHCO_3_ (5 mM) pH 7.3] and incubated in PSS at room temperature (15 min). Measurements were performed at 470–495 nm excitation and 515–575 nm emission and analysed using ScreenWorks Software (v2.0.0.27, Molecular Devices).

### Overexpression of Ca_v_3.2 (*CACNA1H*)

SKBR3 cells were transfected in 6-well plates (375 000 cells/well) 24 h after seeding using Lipofectamine^®^3000 and PC3000 enhancer (Life Technologies) with 2.5 µg of total DNA [pCDNA3.1 (Invitrogen) + pEGFP-N1 (Clontech)] (EGFP MOCK) or (α1Ha (Adgene, 45809, Cambridge, USA) + pEGFP-N1) (EGFP Ca_v_3.2) at equimolar ratio of 10:1. After transfection (24 h) cells were sorted into EGFP enriched (>90 % purity) and EGFP depleted populations using a MoFlo Astrios FACS, RNA was isolated and the effect on expression of selected markers was analysed using RT-qPCR.

### Relative *CACNA1H* (Ca_v_3.2) expression levels in breast cancer subtypes

The relative expression levels of *CACNA1H* were analysed in the cancer genome atlas network (TCGA) [35] breast cancer data set. Expression levels were defined as log2 transformed mean-centred transcript quantification produced by the TCGA consortium through RSEM (RNA-seq by expectation–maximization) [36]. TCGA tumours were divided into quartiles of *CACNA1H* expression. *ERBB2*, *ESR1* and *PGR* levels were compared in each quartile to the quartile with the lowest level of *CACNA1H* expression.

### Assessment of patient survival and response to chemotherapy (CT) in oestrogen receptor positive (ER+), HER2-positive (HER+) and triple negative (TNBC) tumours based on *CACNA1H* expression

Receiver operator curve (ROC) analysis was performed in each clinical subgroup and within the METABRIC [37] cohort that received chemotherapy (CT). The variable for ROC analysis was *CACNA1H* expression while the classification variable was patient survival (1 = death from breast cancer, 0 = non-event). The optimal criterion, as determined by MedCalc (https://www.medcalc.org/) factoring in disease prevalence as death caused by breast cancer, was used to stratify tumours into high and low expressing groups. Groups were used for stratifying patients by overall survival using Kaplan–Meier curves. The online tool KM-Plotter [38] was used to generate a validation set of Kaplan–Meier curves based on *CACNA1H* expression. Survival of patients was stratified using the “Auto select best cutoff” feature, which determines the optimal patient stratification based on median, tertile and quartile groupings. Logrank P values and hazard ratios were determined using MedCalc. Hazard ratios with corresponding 95 % confidence interval and P values are as indicated.

### Statistical analyses

GraphPad Prism (v6.05 for Windows; GraphPad Software, Inc., La Jolla, CA, USA) was used to determine statistical differences.

## Results

### Development and characterisation of trastuzumab-resistant SKBR3 cell lines

**Fig. 1:**
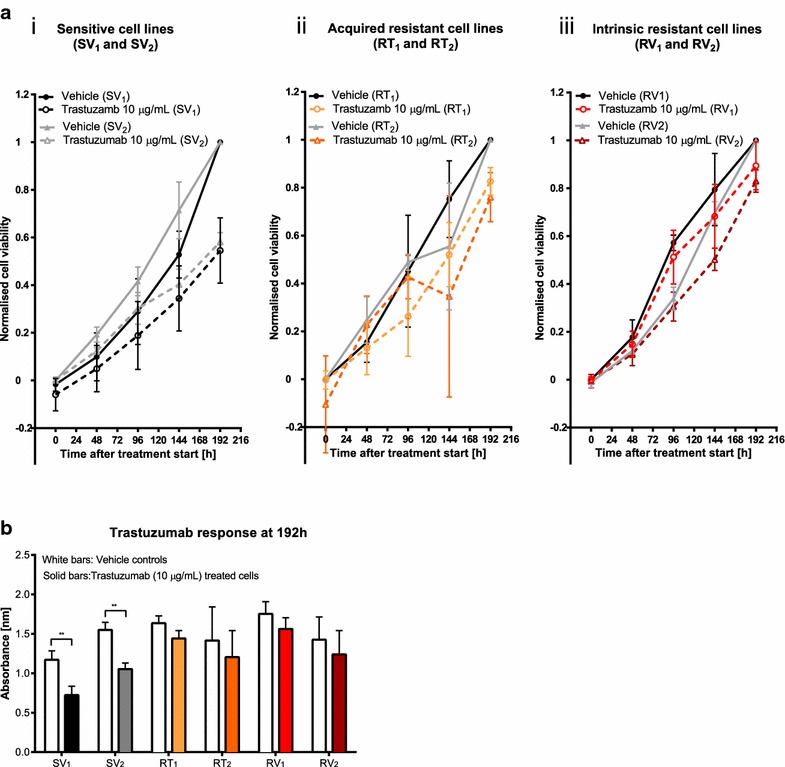
Development of SKBR3-derived cell lines as in vitro models for acquired and intrinsic drug-resistance. SKBR3 cells were continuously cultured for 7 months in the absence or the presence of 10 µg/mL trastuzumab. **a** Cells were seeded at a density of 2000 cells/well and treated with 10 µg/mL trastuzumab or equal volume of H_2_O (vehicle), 24 h after seeding and relative cell viability was assessed over 192 h with a MTS assay. Two cell lines continuously cultured in the absence of trastuzumab (SV_1_, SV_2_) retained trastuzumab-sensitivity (*i*), two cell lines cultured in the presence of trastuzumab (RT_1_, RT_2_) acquired trastuzumab-resistance (*ii*) and two cell lines continuously cultured in the absence of trastuzumab (RV_1_, RV_2_) developed intrinsic resistance to trastuzumab (*iii*). Viable cell number of all samples are expressed relative to control cells (no trastuzumab) at 192 h. Normalised cell viability = [Absorbance of sample (t) − Average absorbance of control (t = 0)]/[Average absorbance control (t = 192 h) − Average absorbance control (t = 0)], (n = 3 ± SD). **b** Relative viable cell numbers of all cell lines were compared at the end of the protocol (192 h after start of treatment). Trastuzumab sensitive cell lines (SV_1_, SV_2_) showed a significant decreased cell viability in the presence of trastuzumab compared to vehicle control cells, whereas the relative viable cell number of acquired resistant cell lines (RT_1_, RT_2_) and intrinsic resistant cell lines (RV_1_, RV_2_) was not significantly altered by trastuzumab (n = 3 ± SD). Statistical analyses were performed using multiple t tests with Holm Sidak correction (**p ≤ 0.01). White bars present vehicle controls and solid coloured bars represent trastuzumab (10 µg/mL) treated cells

Trastuzumab-resistant cell lines and sensitive age-matched control cell lines were established from trastuzumab-sensitive parental SKBR3 cells by continuous culture in trastuzumab (10 µg/mL) for 7 months. Cell lines were assigned as “T” for continuous culture in trastuzumab or “V” for vehicle, “S” for sensitive and “R” for resistant. Cell line status after the 7 months of culture was analysed in the presence of trastuzumab (10 µg/mL) over 192 h (Fig. 1a). Most cell lines (6 of 8) cultured in the absence of trastuzumab retained sensitivity e.g. [SV_1_, SV_2_, Fig. 1a (i)]. Two cell lines cultured in the presence of trastuzumab were selected for further study based on the acquisition of trastuzumab-resistance. In these two cell lines the relative cell viability was not decreased by trastuzumab treatment compared to vehicle controls [RT_1_, RT_2_, Fig. 1a (ii)]. Two of eight age-matched control cell lines cultured continuously for 7 months in the absence of trastuzumab spontaneously developed resistance to trastuzumab and were defined as exhibiting intrinsic resistance to trastuzumab [RV_1_, RV_2_, Fig. 1a (iii)]. Trastuzumab-sensitive cell lines (SV_1_ and SV_2_) showed a significant decrease in cell viability after trastuzumab treatment, while acquired (RT_1_ and RT_2_) and intrinsic (RV_1_ and RV_2_) resistant cells showed no significant difference compared to vehicle controls (**p ≤ 0.1) (Fig. 1b).

### Assessment of HER2 and EGFR expression in trastuzumab-resistant SKBR3 cell lines

**Fig. 2:**
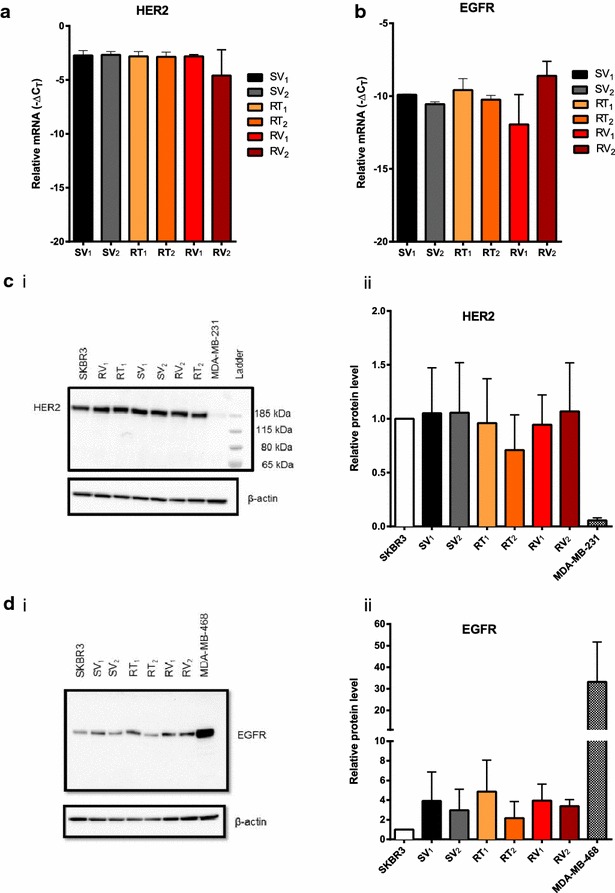
Sensitive and resistant SKBR3-derived cell lines retained their HER2 and EGFR expression levels. Assessment of mRNA levels in SKBR3-derived cell lines showed no differences in the mRNA levels of HER2 (**a**) and EGFR (**b**) in trastuzumab-resistant cell lines (RT_1_, RT_2_, RV_1_, RV_2_) compared to sensitive age-matched control cell lines (SV_1_, SV_2_). All mRNA levels were normalised to 18S rRNA and values expressed as −ΔC_T_ (n = 3 ± SD). The protein level of HER2 (**c**) and EGFR (**d**) were analysed in all SKBR3-derived cell lines and representative immunoblots are shown in (**c** *i*) and **d** *i*). All protein levels were normalised to β-actin protein expression, parental SKBR3 cells were used to assess changes in protein levels through continuous culture, MDA-MB-231 cells were used as a HER2-negative control (**c**) and MDA-MB-468 as an EGFR positive control (**d**). HER2 (**c** *ii*) and EGFR (**d** *ii*) protein expression was normalised to the SKBR3 parental cell line in biological replicates (n = 3 ± SD). Sensitive and resistant SKBR3-derived cell lines showed similar levels of EGFR and HER2 protein expression. Statistical analyses were performed using one-way ANOVA with Bonferroni post-tests (*p* > 0.05)

To confirm that trastuzumab-resistance amongst the model cell lines was not due to loss of HER2 expression, or alterations in EGFR expression, mRNA and protein levels of HER2 and EGFR were quantified in all cell lines (Fig. 2). Resistant cell lines showed similar levels of HER2 and EGFR mRNA compared to trastuzumab-sensitive cell lines (*p* > 0.5) (Fig. 2a, b). HER2 protein (185 kDa) was seen in all samples, with no truncated receptor detected. HER2 protein levels were similar in all SKBR3-derived cell lines compared to parental SKBR3 cells and no differences in protein levels were observed between resistant and sensitive cell lines (*p* > 0.5) (Fig. 2c). EGFR protein levels were also similar in sensitive and resistant cell lines (Fig. 2d). Collectively these data suggested that the mechanism of resistance in this model was not related solely to changes in the expression of HER2 or EGFR.

### Assessment of mRNA levels of calcium channels, pumps and regulating proteins identifies elevated Ca_v_3.2 in trastuzumab-resistant cell lines

**Fig. 3:**
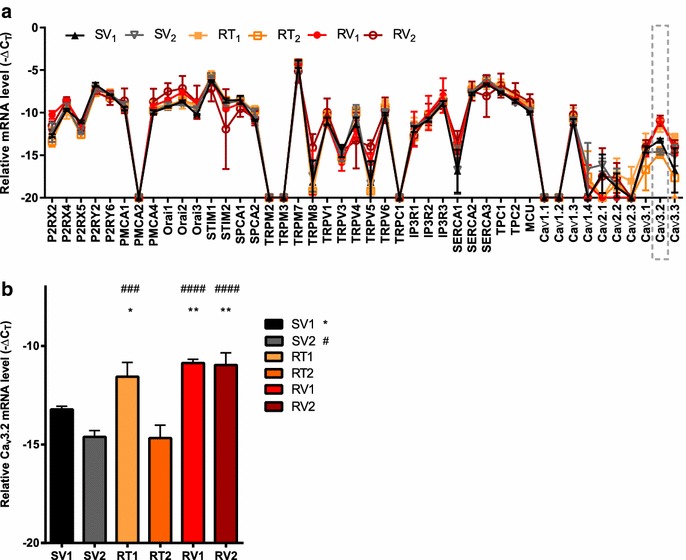
Ca_v_3.2 upregulation in trastuzumab-resistant cell lines. **a** Assessment of mRNA levels of 44 calcium pumps, channels and calcium regulating proteins identified increased expression of Ca_v_3.2 in three of four trastuzumab resistant cell lines. All values were measured relative to 18 S rRNA and expressed as −ΔC_T_. Data points at Y = −20 represent no mRNA detection (n = 3 ± SD). **b** Acquired resistant cell line RT_1_ and both intrinsic resistant cell lines (RV_1_, RV_2_) showed significantly increased expression of Ca_v_3.2 compared to trastuzumab sensitive age-matched control (SV_1_) (n = 3 ± SD). Statistical analyses were performed using one-way ANOVA with Bonferroni post-tests (*p ≤ 0.05, **p ≤ 0.01)

Assessment of over 40 targets including purinergic receptors (P2RX2/4/5, P2RY2/6), calcium pumps (PMCAs, SPCAs, SERCAs) and calcium permeable ion channels (Orai, TRPs, IP3Rs) demonstrated similar mRNA levels of most targets between trastuzumab-sensitive and resistant cell lines, except for the voltage gated calcium channel Ca_v_3.2 (Fig. 3a). Further analysis confirmed the elevation of Ca_v_3.2 mRNA in the RV_1_, RV_2_ and RT_1_ resistant cell lines in comparison to both sensitive cell lines SV_1_ and SV_2_ (Fig. 3b).

### Pharmacological inhibition of Ca_v_3.2 does not restore trastuzumab-sensitivity in trastuzumab-resistant SKBR3 cells

**Fig. 4:**
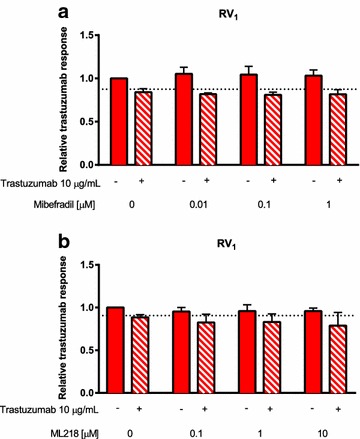
The effect of pharmacological inhibition of Ca_v_3.2 on trastuzumab-resistance. **a** Cells were treated with mibefradil (0.01–1 µM) alone or with trastuzumab (10 µg/mL) 24 h after seeding for 192 h (n = 3 ± SD). Mibefradil did not promote the response to trastuzumab (*p* > 0.05). **b** Cells were treated with ML218 (0.1–10 µM) alone or with trastuzumab (10 µg/mL) 24 h after seeding for 192 h (n = 3 ± SD). ML218 did not promote the response to trastuzumab (*p* > 0.05). Statistical analyses were performed using two-way ANOVA with Bonferroni post-tests

Ca_v_3.2 inhibition was assessed in the trastuzumab-resistant cell line (RV_1_), which had the most pronounced upregulation of Ca_v_3.2 relative to trastuzumab-sensitive SKBR3 cell lines. Cells were treated with mibefradil, a calcium channel blocker, which inhibits both T-type and L-type voltage-gated calcium channels, but with greater effectiveness for T-type calcium channel inhibition [33]. Treatment with mibefradil (0.01–1 µM) did not enhance the trastuzumab (10 µg/mL) response in the RV_1_ resistant cell line (Fig. 4a). Treatment with ML218, a pharmacological inhibitor of Ca_v_3.1, Ca_v_3.2 and Ca_v_3.3 channels with higher selectivity for Ca_v_3.2 [34] at 0.1–10 µM also did not enhance the trastuzumab (10 µg/mL) response in the RV_1_ cell line (Fig. 4b). Thus pharmacological inhibition of the Ca_v_3.2 calcium channel with the inhibitors used in this study did not restore sensitivity to trastuzumab.

### Assessment of ATP-mediated alterations in [Ca^2+^]_CYT_ in trastuzumab-sensitive and resistant SKBR3 derived cell lines

**Fig. 5:**
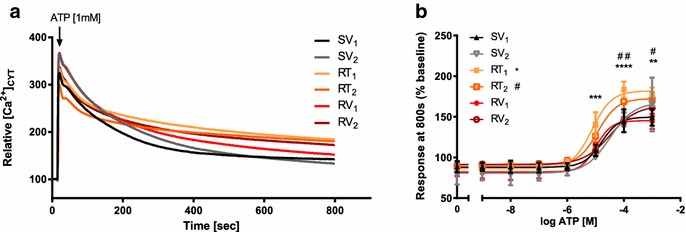
Intracellular calcium measurements with ATP stimulation in trastuzumab-sensitive and resistant cell lines. **a** Example of relative cytosolic calcium [Ca^2+^]_CYT_ responses after ATP [1 mM] stimulation in trastuzumab-sensitive age-matched controls (SV_1_, SV_2_), acquired resistant cell lines (RT_1_, RT_2_) and intrinsic resistant cell lines (RV_1_, RV_2_). **b** Relative calcium [Ca^2+^]_CYT_ responses at 800 s with ATP stimulation [1 nM to 1 mM] in aged-matched control and resistant cell lines (n = 3 ± SD). Acquired resistant cell lines (RT_1_, RT_2_) showed a slower recovery after ATP stimulation with a significant higher relative calcium [Ca^2+^]_CYT_ level at 800 s while intrinsic resistant cell lines (RV_1_, RV_2_,) showed similar recovery rate and similar relative calcium [Ca^2+^]_CYT_ levels compared to aged matched control SV_1_. Statistical analysis was performed using two-way ANOVA with Bonferroni post-tests, compared to SV_1_ (*p ≤ 0.05, **p ≤ 0.01, ***p ≤ 0.001, ****p ≤ 0.0001)

To assess if Ca^2+^-signalling alterations were associated with trastuzumab-resistance, [Ca^2+^]_CYT_ in response to purinergic receptor activation with ATP (1 mM) was measured. Some resistant cell lines appeared to have a delayed recovery in [Ca^2+^]_CYT_ after ATP stimulation (Fig. 5a). ATP concentration–response curves (1 nM to 1 mM) were used to characterise changes in intracellular Ca^2+^-signalling. The relative [Ca^2+^]_CYT_ at 800 s, a measure of recovery after ATP stimulation, was higher in acquired trastuzumab-resistant SKBR3 cell lines (RT_1_ and RT_2_) compared to trastuzumab-sensitive age-matched controls (Fig. 5b).

### Overexpression of Ca_v_3.2 in parental SKBR3 cells does not increase expression of selected mRNA markers associated with drug-resistance

**Fig. 6:**
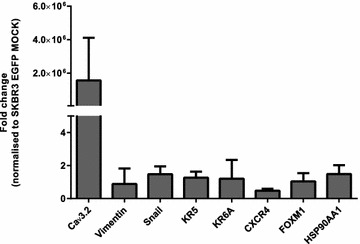
Effect of *CACNA1H* (Ca_v_3.2) overexpression on expression of mRNA markers associated with tumour progression and drug-resistance. SKBR3 parental cells were co-transfected with α1Ha + pEGFP-N1 (EGFP Ca_v_3.2) or pCDNA3.1 + pEGFP-N1 (EGFP MOCK) and sorted into EGFP enriched and depleted populations 24 h after transfection. Results are expressed as fold change normalised to EGFP MOCK. EGFP Ca_v_3.2 cells showed an approximate greater than 2 10^6^ fold increase in expression compared to EGFP MOCK cells (n = 3 ±SD). Overexpression of Ca_v_3.2 did not increase levels of markers associated with EMT (*vimentin*, *snail*), basal markers (*KR5*, *KR6*A ) and markers associated with drug-resistance (*CXCR4*, *FOXM1*, *HSP90AA1*) (n = 3 ±SD)

Since Ca_v_3.2 is upregulated in trastuzumab-resistant SKBR3 cells and given the role of Ca^2+^-signalling in the activation of transcription factors [39] we hypothesised that Ca_v_3.2 may induce the expression of genes related to therapeutic resistance and/or with cellular phenotypes associated with therapeutic resistance [40–44]. SKBR3 parental cells were either co-transfected with pEGFP-N1 + α1Ha (EGFP Ca_v_3.2) or with pEGFP-N1  + the empty plasmid backbone (EGFP MOCK) as a control (Fig. 6). Overexpression of Ca_v_3.2 did not produce any pronounced increase of the mRNA levels of *vimentin, snail, KRT5, KRT6A, CXCR4, FOXM1* or *HSP90AA1* (Fig. 6).

### Assessment of Ca_v_3.2 expression levels in cell lines and clinical breast cancer molecular subtypes

**Fig. 7:**
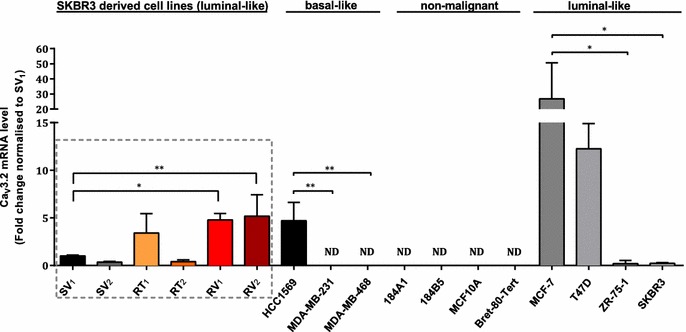
Assessment of the expression level of *CACNA1H* (Ca_v_3.2) in SKBR3 derived trastuzumab-resistant cell lines, different breast cancer cell lines and non-malignant breast cell lines. Ca_v_3.2 was not detected (ND) in non-malignant breast cell lines and the basal breast cancer cell lines MDA-MB-231 and MDA-MB-468. The luminal cell lines MCF-7 and T47D showed the highest expression of Ca_v_3.2. Statistical analysis was performed within each subtype and trastuzumab-resistant cell lines were compared to the SV_1_ trastuzumab sensitive SKBR3 cell line. For all amplified targets (n = 3 ±SD), statistical analysis was performed using one-way ANOVA with Bonferroni post-test (*p ≤ 0.05, **p ≤ 0.01)

Levels of Ca_v_3.2 mRNA were then assessed in a panel of non-malignant breast and breast cancer cell lines representing different molecular subtypes (Fig. 7). Ca_v_3.2 mRNA levels in three of the trastuzumab-resistant cell lines (RT_1_, RV_1_ and RV_2_) were similar to the basal-like HER2 overexpressing trastuzumab-resistant cell line HCC1569 [45]. Basal-like breast cancer cell lines (MDA-MB-231, MDA-MB-468) had undetectable levels of Ca_v_3.2 mRNA, except for HCC1569 cells. In non-malignant breast cell lines (184A1, 184B5, MCF10A, Bret-80-Tert) Ca_v_3.2 mRNA was not detected. Highest levels of Ca_v_3.2 were seen in the luminal-like breast cancer lines MCF-7 and T47D. However, Ca_v_3.2 mRNA levels were dramatically different between luminal-like breast cancer cell lines, with very low levels in ZR-75-1 and parental SKBR3 cell lines and very high levels in MCF-7 and T47D (Fig. 7).

**Fig. 8:**
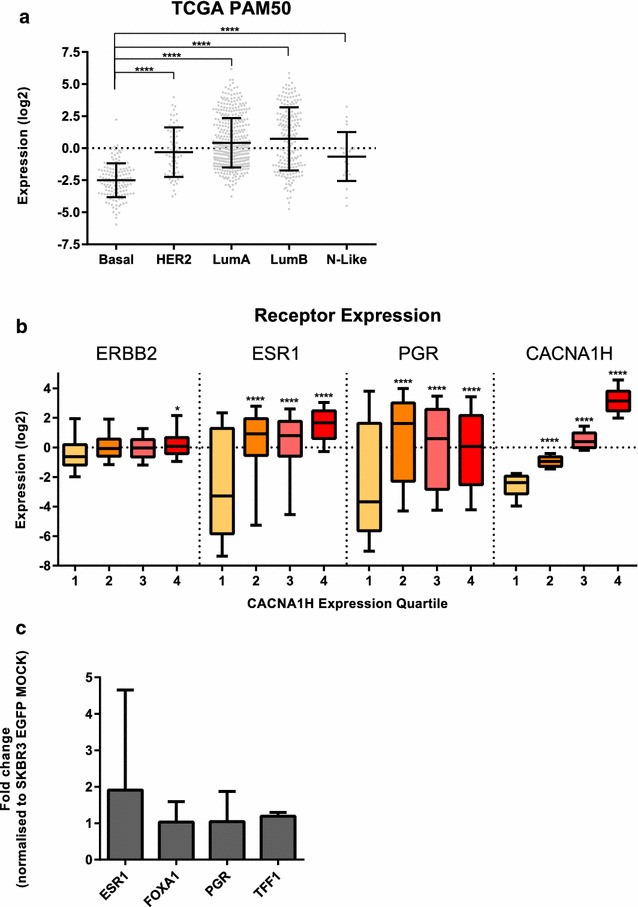
**a** Analysis of *CACNA1H* (Ca_v_3.2) expression across the intrinsic molecular subtypes of breast cancer in clinical patient samples. The relative *CACNA1H* expression level (log2 normalised) was produced by the TCGA consortium through RSEM [36]. The TCGA tumour cohort consists of 845 tumours with 140 basal-like (Basal), 67 HER2-enriched (HER2), 420 luminal A (LumA), 194 luminal B (LumB) and 24 normal-like (N-Like) as determined by RNA-Seq based PAM50 allocations by the TCGA consortium. The PAM50 intrinsic molecular subtypes are based on the classifications of gene expression patterns previously described [66, 67]. This classification was performed by the TCGA consortium [35]. Horizontal lines represent data means with standard deviation and data points in grey. Statistical analysis was performed on expression levels of *CACNA1H* in basal-like compared to HER2-enriched and luminal subtypes using a one-way ANOVA with Sidak corrected multiple comparisons,(****p ≤ 0.0001). **b** Relative gene expression for breast cancer receptors *ERBB2* (HER2), *ESR1* (oestrogen receptor) and *PGR* (progesterone receptor) within each quartile of *CACNA1H* expression. Statistical analysis was performed using a one-way ANOVA with Sidak corrected multiple comparisons, comparing expression levels between the highest and the lowest quartile for each gene (*p ≤ 0.05, ****p ≤ 0.0001). **c** Ca_v_3.2 overexpression in SKBR cells (SKBR3 EGFP) did not increase expression of hormone receptors or proteins involved in oestrogen-receptor mediated signalling *TFF1*, *FOXA1*, quantified using RT-qPCR. Results are expressed as fold change normalised to EGFP MOCK (n = 3 ±SD)

Ca_v_3.2 expression was also assessed in clinical breast cancer samples stratified into the intrinsic molecular subtypes using the cancer genome atlas expression data (TCGA) [35] (Fig. 8a). The luminal A and B subtypes showed the highest expression level of *CACNA1H* (Ca_v_3.2), with the basal subtype expressing significantly lower levels of Ca_v_3.2 compared to the luminal A, B and HER2 subtypes. Consistent with the cell line data, luminal breast cancers were associated with a wide range of Ca_v_3.2 levels.

### High levels of Ca_v_3.2 are associated with increased levels of *ESR1* and *PGR* receptor but Ca_v_3.2 itself does not induce hormone receptor expression or expression of luminal markers in SKBR3 cells

Given the strong association between Ca_v_3.2 and the luminal A and B molecular subtypes (Fig. 8a), we also assessed hormone receptor levels in different *CACNA1H* (Ca_v_3.2) expression quartiles using the TCGA database, to address the hypothesis that this may reflect a direct regulatory effect. Breast cancers with high levels of *CACNA1H* (Ca_v_3.2) (2nd, 3rd, 4th quartile) showed a significant elevation in levels of *ESR1* and *PGR* compared to breast cancers with significantly low levels of Ca_v_3.2 (1st quartile) (Fig. 8b). A significant correlation between *ERBB2* was also observed, however, only minor compared to *ESR1* and *PGR* (Fig. 8b). To define a potential role for Ca_v_3.2 in the expression of *ESR1* and *PGR*, we analysed the expression levels of *ESR1*, *FOXA1*, *PGR* and *TFF1* in SKBR3 cells with induced overexpression of Ca_v_3.2 (EGFP Ca_v_3.2 SKBR3 cells). These results indicated that Ca_v_3.2 is not a regulator of the expression of *ESR1* and *PGR* or the luminal markers* FOXA1* and *TFF1* (Fig. 8c).

### High *CACNA1H* (Ca_v_3.2) expression levels in ER-positive (ER+) breast cancer are associated with poor prognosis, whereas in HER2-positive (HER2+) breast cancer patients it is associated with better responses to chemotherapy (CT)

**Fig. 9:**
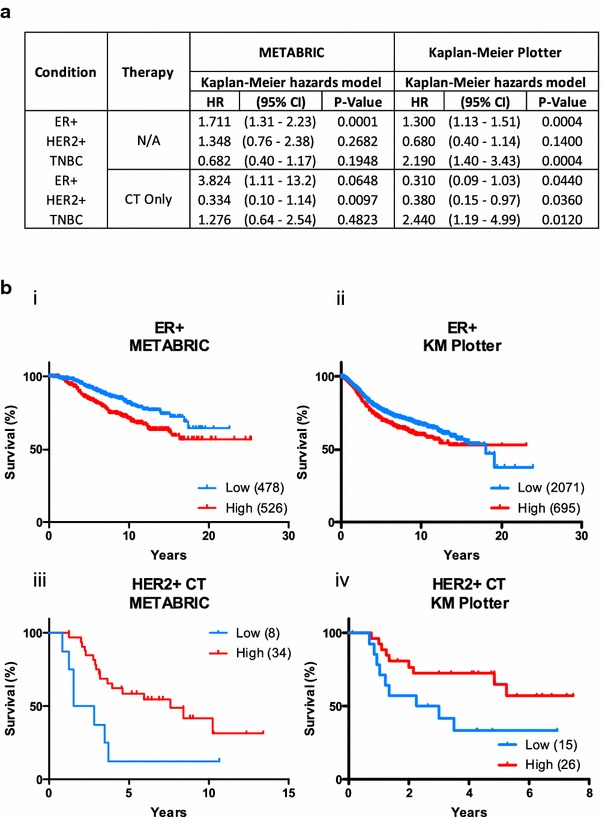
Ca_v_3.2 expression stratifies the survival of breast cancer patients. The survival of patients from both the METABRIC and Kaplan–Meier Plotter cohorts were stratified based on the expression of Ca_v_3.2 for each of the clinical subgroups and within those groups, in patients treated with chemotherapy (CT). METABRIC patients were stratified on overall survival (OS) and the KM-Plotter cohort on relapse free survival (RFS). Log-rank hazard ratios (HR) and corresponding P values are shown (**a**). Kaplan–Meier curves from data shown in panel A for ER+ patients (**b** *i*, *ii*) and those with HER2-positive tumours (HER+) treated with CT (**b** *iii*, *iv*). Tumours were stratified on the basis of their *CACNA1H* expression into low and high expressing groups, numbers indicated in brackets

Given the association between Ca_v_3.2 and trastuzumab-resistance in HER2-positive breast cancer cell lines in vitro and increased expression within the luminal subtype, we explored the potential of Ca_v_3.2 as a biomarker in predicting patient survival and/or outcomes with chemotherapy. The overall survival of patients from both METABRIC [37] and KM-Plotter [38] cohorts were analysed based on their expression level of *CACNA1H* (Ca_v_3.2) (high/low) in each subtype and within each group for patients receiving chemotherapy (CT) (Fig. 9a). Our analysis identified that high levels of Ca_v_3.2 are associated with poor survival in ER + tumours in METABRIC and KM-Plotter cohorts [Fig. 9a, b (i, ii)]. Note that beyond 20 years, survival of ER+ patients appear to be better for those with high expression, however, due to a small sample size remaining in the study at this time (9 patients) we were unable to explore this further. In all other subtypes, expression levels of Ca_v_3.2 were not consistently stratified with overall survival in both cohorts (Fig. 9a). We then analysed the treatment response from the METABRIC and KM-Plotter cohorts towards chemotherapy in each subgroup. Interestingly, this analysis identified that HER2-positive patients receiving chemotherapy with tumours expressing high levels of *CACNA1H* demonstrated a better overall survival after chemotherapy, compared to patients with low levels of *CACNA1H* in both, METABRIC and KM-Plotter cohorts [Fig. 9a, b (iii, iv)]. In all other subtypes, Ca_v_3.2 did not significantly affect survival with therapy in both cohorts (Fig. 9a).

## Discussion

Our work explores alterations in the expression of calcium channels in the context of trastuzumab-resistance and therapeutic response. The work presented here demonstrated that levels of Ca_v_3.2, a voltage gated T-type calcium channel, are elevated in acquired and intrinsic SKBR3 breast cancer cell line models of trastuzumab-resistance. Whilst we found no evidence for a direct role of Ca_v_3.2 in driving or reversing trastuzumab-resistance, our data suggest that Ca_v_3.2 may be an informative prognosis marker in ER+ breast cancer patients for overall survival and in HER2-positive breast cancer patients with chemotherapy.

In vitro models for trastuzumab-resistance include cell lines representing intrinsic resistance such as those established from the breast cancers of patients that do not respond to trastuzumab therapy despite HER2 overexpression [46], and those that represent acquired resistance, established through continuous culture of HER2-positive breast cancer cell lines in the presence of trastuzumab [31, 47]. Intrinsic resistance and acquired resistance may be mediated through different mechanisms [10]. In this study we developed a model system derived from the same cell line, suitable for assessing the mechanistic pathways that may be responsible for spontaneous (intrinsic) and acquired resistance derived from the same cell line. Intrinsic trastuzumab-resistant SKBR3 cell lines were established during the production of age-matched controls, which was not unexpected as continuous culturing can have a major impact on cellular phenotypes [48, 49].

The dysregulation of calcium homeostasis and the expression levels of specific calcium channels and pumps can be a feature of tumour progression [26, 50, 51] and is associated with the acquisition of resistance to adriamycin, the IGF-1 receptor inhibitor ganitumab and other agents [29, 52]. Our assessment of a wide panel of calcium pumps, channels and channel modulators identified an upregulation of the voltage gated Ca^2+^ channel Ca_v_3.2 in three of four trastuzumab-resistant SKBR3 cell lines. Consistent with the variety of pathways associated with trastuzumab resistance [10, 16, 18], Ca_v_3.2 was not elevated in all of the resistant cell lines, with elevated Ca_v_3.2 absent from RT_2_ cells. Ca_v_3.2 is a T-type voltage-gated calcium channel [53] generally expressed in cells of the heart, brain and liver and some tumours [53, 54]. High expression levels of T-type channels are associated with increased proliferation in breast cancer cells [55] and tumour progression in prostate cancer cells [56]. Ca_v_3.2 is also proposed as an oncogene candidate in T cell leukaemia [57]. A higher level of Ca_v_3.2 was also identified in the basal-like, HER2-positive, intrinsically trastuzumab-resistant cell line HCC1569. Intrinsic resistance to trastuzumab is associated with expression of basal markers [58]. Thus Ca_v_3.2 expression might be a feature of breast cancers that are trastuzumab-resistant and also express basal markers. Our studies also demonstrated that although Ca_v_3.2 may be a marker for activation of some trastuzumab-resistance pathways, inhibition of Ca_v_3.2 is unable to restore trastuzumab-sensitivity in SKBR3 cell lines with acquired or intrinsic trastuzumab-resistance. Ca_v_3.2 inhibitors therefore do not appear to represent an effective way to overcome trastuzumab-resistance in HER2-positive breast tumours. Although induced overexpression did not promote expression of resistance markers, future studies using T-type Ca^2+^ channel activators when available should assess the effect of these agents on the expression of resistance markers and trastuzumab resistance, analogous to studies which have evaluated the effects of pharmacological activation of L-type Ca^2+^ channels with (S)-(-)-Bay K 8644 [59, 60]. Only the acquired trastuzumab-resistant cell lines RT_1_ and RT_2_ showed a different calcium response after ATP stimulation compared to trastuzumab-sensitive controls, resulting in a delayed recovery. Although the response to ATP showed only minor alterations between sensitive and resistant cell lines and was not observed in all resistant cell lines, Ca_v_3.2 might be involved in activation of transcription factors as previously shown for other voltage gated calcium channels [39]. However, our studies identified that Ca_v_3.2 is not a driver of basal, epithelial to mesenchymal (EMT) transition or markers associated with resistance in SKBR3 cells. Ca_v_3.2 mRNA was highly expressed in some luminal-like breast cancer cell lines (MCF-7 and T47D) compared to basal breast cancer cell lines that lack HER2 amplification, and was undetectable in cell lines derived from non-malignant breast tissue. The relationship between Ca_v_3.2 and the luminal subtype was also observed in clinical breast cancer samples, with significantly higher Ca_v_3.2 levels in luminal A and B subtypes compared to basal breast cancers. Although not a driver for *ESR1* and *PGR* levels in SKBR3 cells, there was a strong correlation between Ca_v_3.2 and levels of these hormone receptors in breast cancers. Analyses of clinical data showed that high levels of Ca_v_3.2 were associated with poor prognosis in ER+ breast cancer patients. Surprisingly, our studies identified that high levels of Ca_v_3.2 was associated with a better response to chemotherapy in patients with HER2-positive breast cancers.

The apparent contradiction of Ca_v_3.2 mRNA levels being negatively associated with survival in ER+ cancers and positively associated with survival with chemotherapy in HER-positive breast cancers, may be reflective not only of potential differential contribution of calcium signalling in different breast cancer subtypes but also in the diversity of the Ca^2+^-signal. Some aspects of calcium signalling could be quite different between HER2+ and ER+ breast cancers and this could contribute to differences in the association between Ca_v_3.2 levels and survival, for example Orai3-mediated Ca^2+^-influx is Ca^2+^-store dependent in ER positive but not ER negative breast cancer cells lines [61]. Ca^2+^-influx can promote cellular proliferation or be an inducer of cell death [50]. In the context of the association between high Ca_v_3.2 levels and poor prognosis in ER+ breast cancers, Ca_v_3.2-mediated constitutive Ca^2+^-influx contributes to enhanced proliferation in the LNCaP prostate cancer cell line [62]. Whereas the association between high levels of Ca_v_3.2 and better outcomes with chemotherapy in HER2-positive breast cancer may be related to important role of Ca^2+^-increases during cell death [63]. Although not yet studied in HER2-positive breast cancer cell lines, the T-type Ca^2+^-channel Ca_v_3.1 is a key mechanism by which cyclophosphamide induces apoptosis in luminal MCF-7 breast cancer cells [64] and Ca_v_3.2 is essential in the inhibitory effects of epigallocatechin-3-gallate on the viability of MCF-7 breast cancer cells [65].

## Conclusion

Our data suggest that at the clinical level, Ca_v_3.2 may be similarly important in the responses to chemotherapy in HER2-positive breast cancers, however, further studies are required. In summary, these studies have identified enhanced expression of Ca_v_3.2 as a feature of trastuzumab-resistant breast cancer cells, however, Ca_v_3.2 does not seem to be a driver of trastuzumab-resistance. Further studies are now required to elucidate the potential differential roles of Ca_v_3.2 in different breast cancer subtypes and its utility as a potential biomarker of prognosis and therapeutic responsiveness in patients with ER + and HER2-positive breast cancers respectively.

## Additional file

10.1186/s12935-016-0299-0 Gene expression assays used in these studies to assess relative mRNA levels of target genes.

## Abbreviations

CT: chemotherapy
EMT: epithelial to mesenchymal transition
ER+: oestrogen receptor positive
HER2: human epidermal growth factor 2
ROC: receiver operator curve
RSEM: RNA seq by expectation maximization
RT-qPCR: real time quantitative polymerase chain reaction
TCGA: the cancer genome atlas network
TNBC: triple negative breast cancer
