# Supplementary material for: The voltage gated Ca2+-channel Cav3.2 and therapeutic responses in breast cancer
Source: Cancer Cell Int. 2016 Mar 31;16:24. doi: 10.1186/s12935-016-0299-0 (PMC4815142; doi:10.1186/s12935-016-0299-0)
Supplement: Supplementary file 1 — 10.1186/s12935-016-0299-0 Gene expression assays used in these studies to assess relative mRNA levels of target genes. [file 12935_2016_299_MOESM1_ESM.docx]

**Data S1:**

The following gene expression assays (Life technologies) were used in these studies to assess relative mRNA levels of target genes**:**

EGFR (Hs01076092_m1), HER2 (Hs00170433_m1), P2RX2 (Hs04176268_g1), P2RX4 (Hs00602442_m1), P2RX5 (Hs01112471_m1), P2RY2 (Hs04176264_s1), P2RY6 (Hs00602548_m1), PMCA1 (Hs00155949_m1), PMCA2 (Hs00155975_m1), PMCA4 (Hs00608066_m1), Orai1 (Hs00385627_m1), Orai2 (Hs00259863_m1), Orai3 (Hs00743683_s1), STIM1 (Hs00162394_m1), STIM2 (Hs00372712_m1), SPCA1 (Hs00205122_m1), SPCA2 (Hs00208296_m1), TRPM2 (Hs01066085_m1), TRPM3 (Hs00257553_m1), TRPM7 (Hs00292383_m1), TRPM8 (Hs00375481_m1), TRPV1 (Hs00218912_m1), TRPV2 (HS00901640_m1), TRPV3 (Hs00376854_m1), TRPV4 (Hs01099348_m1), TRPV5 (Hs00219765_m1), TRPV7 (Hs00367960_m1), TRPC1 (Hs01553152_m1), IP3R1 (Hs00181881_m1), IP3R2 (Hs00181916_m1), IP3R3 (Hs01573555_m1), SERCA1 (Hs01092295_m1), SERCA2 (Hs00544877_m1), SERCA3 (Hs00193090_m1), TPC1 (Hs00330542_m1), TPC2 (Hs01552063_m1), MCU (Hs00293548_m1), Ca_v_1.1 (Hs01374398_m1), Ca_v_1.2 (Hs00167681), Ca_v_1.3 (Hs01073321_m1), Ca_v_2.1 (Hs01579431_m1), Ca_v_2.2 (Hs01053090_m1), Ca_v_2.3 (Hs00167789_m1), Ca_v_3.1 (Hs00367969_m1), Ca_v_3.2 (Hs00234934_m1), Ca_v_3.3 (Hs00184168_m1), vimentin (Hs00184484_m1), snail (Hs00195591_m1), KRT5 (Hs00361185_m1), KR6A (Hs01699178_g1), CXCR4 (Hs00237052_m1), FOXM1 (Hs01073586_m1), HSP90AA1 (Hs00743767_sH), FOXA1(Hs04187555_m1), PGR (Hs01556702_m1), TFF1 (Hs00907239_m1), ESR1 (Hs00174860_m1) and Eukaryotic 18S rRNA Endogenous Control (4319413E)
